# Does Empirical Antibiotic Use Improve Outcomes in Ventilated Patients with Pandemic Viral Infection? A Multicentre Retrospective Study

**DOI:** 10.3390/antibiotics14060594

**Published:** 2025-06-08

**Authors:** Elisabeth Papiol, Julen Berrueta, Juan Carlos Ruíz-Rodríguez, Ricard Ferrer, Sara Manrique, Laura Claverias, Alejandro García-Martínez, Pau Orts, Emili Díaz, Rafael Zaragoza, Marco Marotta, María Bodí, Sandra Trefler, Josep Gómez, Ignacio Martín-Loeches, Alejandro Rodríguez

**Affiliations:** 1 Intensive Care Department, Vall d’Hebron Universitary Hospital, 08035 Barcelona, Spain; elisabeth.papiol@vallhebron.cat (E.P.); juancarlos.ruiz@vallhebron.cat (J.C.R.-R.); ricard.ferrer@vallhebron.cat (R.F.); 2 Shock, Organ Dysfunction and Resuscitation Research Group, Vall d’Hebron Research Institute (VHIR), 08035 Barcelona, Spain; 3 Faculty of Medical Sciences, Universitat Autònoma de Barcelona, 08035 Barcelona, Spain; 4 Critical Care Department, Hospital Universitari Joan XXIII, 43005 Tarragona, Spain; juberrueta.hj23.ics@gencat.cat (J.B.); saramanrique@gencat.cat (S.M.); lauraclaverias@gmail.com (L.C.); alejgarcia.hj23.ics@gencat.cat (A.G.-M.); pau.orts@urv.cat (P.O.); mmarottapais@gmail.com (M.M.); mbodi.hj23.ics@gencat.cat (M.B.); sitrefler@yahoo.es (S.T.); 5 Tarragona Health Data Research Working Group (THeDaR), 43005 Tarragona, Spain; 6 Critical Care Department, Hospital Parc Tauli, 08208 Sabadell, Spain; emilio.diaz.santos@gmail.com; 7 Critical Care Department, Hospital Dr. Peset, 46017 Valencia, Spain; zaragoza_raf@gva.es; 8 Medicine Department, Rovira i Virgili University, 43002 Tarragona, Spain; 9 Pere Virgili Health Research Institute, 43005 Tarragona, Spain; 10 Centre for Biomedical Research Network Respiratory Diseases (CIBERES), 43005 Tarragona, Spain; 11 Technical Secretary Hospital Universitari Joan XXIII, 43005 Tarragona, Spain; jgomez.alvarez.hj23.ics@gencat.cat; 12 Department of Anaesthesia and Critical Care, Multidisciplinary Intensive Care Research Organization (MICRO), St James’s University Hospital, Trinity Centre for Health Sciences, D08 NHY5 Dublin, Ireland; drmartinloeches@gmail.com; 13 Basic Sciences Department, Rovira I Virgili University, 43002 Tarragona, Spain

**Keywords:** empirical antibiotic treatment, pandemic viral pneumonia, ventilator-associated pneumonia, ICU mortality, antimicrobial stewardship

## Abstract

**Background:** During the influenza A(H1N1) and COVID-19 pandemics, empirical antibiotic treatment (EAT) was widely administered to critically ill patients despite low rates of confirmed bacterial co-infection (COI). The clinical benefit of this practice remains uncertain and may contradict antimicrobial stewardship principles. **Objective:** To evaluate whether EAT at ICU admission reduces ventilator-associated pneumonia (VAP) incidence or ICU mortality in critically ill patients with pandemic viral pneumonia, stratified by presence of COI. **Methods:** This retrospective analysis combined two national multicentre ICU registries in Spain, including 4197 adult patients requiring invasive mechanical ventilation for influenza A(H1N1) or COVID-19 between 2009 and 2021. Primary outcomes were ICU mortality and VAP incidence. Analyses were stratified by microbiologically confirmed bacterial COI. Propensity score matching, Cox regression, General Linear (GLM), and random forest models were applied. **Results:** Among patients without COI (n = 3543), EAT was not associated with lower ICU mortality (OR = 1.02, 95%CI 0.81–1.28, *p* = 0.87) or VAP (OR = 1.02, 95%CI 0.79–1.39, *p* = 0.89). In patients with confirmed COI (n = 654), appropriate EAT was associated with reduced VAP (17.4% vs. 36.3%, *p* < 0.001) and ICU mortality (38.4% vs. 49.6%, OR = 1.89, 95%CI 1.13–3.14, *p* = 0.03) compared to inappropriate EAT. **Conclusions:** EAT was not associated with a lower incidence of VAP or higher survival rates and could be harmful if administered incorrectly. These findings support a more targeted approach to antibiotic use, guided by microbiology, biomarkers and stewardship principles.

## 1. Introduction

The influenza A(H1N1) and COVID-19 pandemics placed enormous pressure on health systems and were responsible for significant global mortality [1,2,3,4]. Although these pandemics occurred a decade apart, both were marked by widespread empirical use of antibiotics, largely encouraged by recommendations from scientific societies and public health authorities [5,6,7,8,9,10]. This practice persisted despite the consistently low prevalence of confirmed bacterial co-infection (COI)—reported in only 6% to 15% of cases—which raised serious concerns about the appropriateness of antimicrobial use and the resulting contribution to antibiotic resistance [10,11,12,13,14,15,16].

In viral pneumonias, antibiotic therapy is generally not indicated unless there is clear evidence of COI [17]. However, clinical uncertainty often complicates early decision-making, particularly in critically ill patients. In the absence of definitive diagnostic information, many clinicians initiate empirical antibiotic treatment (EAT) at the time of intubation as a precautionary measure. While this approach may appear clinically justifiable, it challenges the principles of antimicrobial stewardship, which emphasize minimizing unnecessary antibiotic use and discontinuing therapy as soon as bacterial infection is reasonably excluded [5,7].

The literature to date offers conflicting perspectives. Some studies suggest that EAT at intubation in COVID-19 patients may be associated with lower rates of pulmonary superinfection and mortality [18]. However, the generalizability of these findings remains uncertain, and other studies have not observed similar benefits [9,19,20]. In fact, some have reported an increased incidence of ventilator-associated pneumonia (VAP) associated with empirical antimicrobial use [21,22,23], reinforcing the need for a more cautious and evidence-based approach.

With dozens of viruses capable of causing pneumonia in humans, differentiating viral from bacterial pneumonia in clinical practice using traditional diagnostic methods can be very difficult. Our group [6,14,15] and others [9,16,24] have investigated the value of procalcitonin (PCT) in determining the presence of COI in pandemic viral pneumonia. Although PCT performs better in influenza than in COVID-19, it has been shown to be useful in aiding the diagnosis of COI and optimizing antimicrobial therapy [6,8,14,15,23]. Rather than recommending indiscriminate antimicrobial use in viral pneumonia, a real effort should be made to determine whether or not bacterial COI is present in patients with pandemic viral infection. In this context, the use of PCT and new rapid molecular diagnostic techniques could be a valid tool for optimizing EAT. In our opinion, antibiotics should be used with caution and discontinued unless the patient’s true need has been established. While the administration of EAT in patients with bacterial COI should be appropriate and early, it is important to ensure that EAT is given to those who really need it and used with extreme caution.

We hypothesized that the administration of EAT in this population is not associated with a reduced incidence of VAP or lower ICU mortality, once COI has been reasonably excluded. To address our hypothesis, we conducted a retrospective study using two large multicentre Spanish ICU databases, encompassing 4197 patients who required mechanical ventilation for acute respiratory failure due to either influenza A(H1N1) or COVID-19. Our primary objective was to evaluate the association between EAT and both VAP occurrence and ICU all-cause mortality, while the secondary objective was to assess the consistency of these findings across the two pandemic contexts and to explore the robustness of the results using both conventional statistical approaches and machine learning techniques.

## 2. Materials and Methods

### 2.1. Study Design

This was a secondary, retrospective observational analysis based on two prospective, multicentre cohort studies conducted in Spain. The analysis followed the Strengthening the Reporting of Observational Studies in Epidemiology (STROBE) guidelines [25].

### 2.2. Setting

Data were obtained from two national registries coordinated by the Spanish Society of Intensive Care Medicine (SEMICYUC). The first dataset, the GETGAG registry [26,27], included patients admitted to 184 ICUs with influenza A(H1N1)pdm09 between June 2009 and June 2019. The second dataset, the COVID-19 registry [15,28], involved 74 ICUs and enrolled patients with SARS-CoV-2 infection between 1 July 2020 and 31 December 2021. Ethical approval was obtained for both registries, with appropriate waivers for informed consent due to the observational nature of the study.

### 2.3. Participants

Eligibility criteria: all adult patients admitted to the ICU for acute respiratory failure due to confirmed influenza A(H1N1) pdm09 or SARS-CoV-2 infection requiring invasive mechanical ventilation on admission were eligible.

Exclusion criteria: patients without invasive mechanical ventilation (IMV) on ICU admission and those with microbiologically confirmed fungal co-infection were excluded.

Final cohort: a total of 4197 patients met inclusion criteria and were included in the analysis.

Follow-up: patients were followed until ICU discharge or death.

### 2.4. Variables

Demographic data, comorbidities, and clinical and laboratory findings were collected during the first 24 h after ICU admission. In addition, the need for IMV and the presence of shock upon ICU admission were recorded. Disease severity was determined using the Acute Physiology and Chronic Health Evaluation II (APACHE II) score [29], while the level of organ dysfunction was determined using the SOFA score [30]. The variables controlled for in the study can be seen in Table 1.

Definitions: co-infection (COI), ventilator-associated pneumonia (VAP), empiric antibiotic treatment (EAT), appropriate EAT (AEAT), inappropriate EAT (IEAT), multidrug resistance (MDR), acute kidney injury (AKI), shock and immunosuppression were defined using standardized criteria (CDC, ERS/ESICM/ESCMID/ALAT, KDIGO) [1,14,31,32,33,34]. The exact meanings of these variables can be found in the Supplementary Materials.

### 2.5. Data Sources and Measurement

Viral infections were confirmed via rt-PCR per IDSA [35] and WHO [36] recommendations. Coinfections were confirmed using CDC microbiological criteria. Standardized forms were used for data collection within each registry. Data consistency and integrity were maintained across participating centres. Diagnostic definitions and laboratory standards were harmonized within each registry.

### 2.6. Bias

To mitigate confounding, propensity score matching was applied for comparisons between groups with and without EAT. Multivariate regression and non-linear modelling (random forest) were used to control for known confounders. Definitions were standardized to reduce classification bias.

### 2.7. Analysis Plan and Statistical Analysis (Figure 1)

First, we performed a descriptive analysis distinguishing between patients with and without EAT on ICU admission. Continuous variables are presented as median and quantiles (Q1–Q3) and categorical variables as numbers (n) and percentages. Chi-square and U-Mann–Whitney tests were used to compare between groups.

Second, we performed a descriptive analysis differentiating patients with and without the presence of COI. Within each of these subgroups, we differentiated between those with and without EAT.

Third, within the subgroup of patients with COI, we examined the impact of appropriate EAT on mortality, development of VAP, ICU and hospital LOS, and IMV days. For this analysis, patients with inappropriate EAT (IEAT) were those with IEAT according to microbiological sensitivity and those without EAT on ICU admission.

Fourth, within the subgroup of patients without COI, to analyse the impact of EAT on the study objectives, and to convert an observational study into a quasi-randomized study, a propensity score matching was performed. After matching, the effect of EAT on all-cause ICU mortality and on the development of VAP was examined by Kaplan–Meier plot and differences were determined by Log Rang test.

In addition, a Cox proportional hazards (COX) and GLM model was used to determine whether EAT was a factor associated with VAP or ICU mortality in multivariate adjusted analysis. The results are expressed as hazard ratio (HR) and its 95% confidence interval (CI) for COX model and as Odds ratio (OR) and its 95%CI for GLM.

Fifth, in addition, to evaluate the impact of EAT on patients without COI, a non-linear regression analysis (random forest-RF) was performed to study whether there are non-linear associations between EAT use and crude mortality or the development of VAP that cannot be evidenced by linear analysis (GLM). The performance of the RF model was evaluated using out-of-bag (OOB) error. We also plotted the importance of the different variables for the model, which is related to the average loss of accuracy and the Gini index for the classification model.

Complete information on the statistical analysis is available in the Supplementary Materials.

Statistical analysis was performed with R statistical software (v 4.4.1) R: The R Project for Statistical Computing (r-project.org).

**Figure 1:**
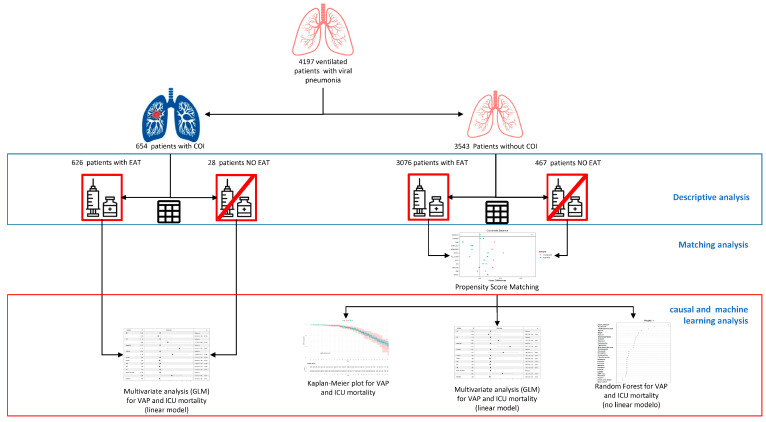
Schematic representation of the statistical analysis performed. A comparative analysis was performed between patients with and without empirical antibiotic treatment (EAT) in the coinfection subgroup (COI). A multivariate analysis was then conducted to identify factors associated with ventilator-associated pneumonia (VAP) and ICU mortality. In the subgroup without COI, a comparative analysis was performed between patients with and without EAT. Propensity score matching was then used to adjust the variables. The impact of EAT on VAP and ICU mortality was then evaluated over time using K-Meier and Cox regression, as a dichotomous variable using linear (GLM) and non-linear (random forest) models.

## 3. Results

### 3.1. Whole Population

A total of 4197 ventilated patients were included in the study (Figure S1). Of these, 3702 (88.2%) received EAT on admission to the ICU. Patients receiving EAT had higher severity of illness, higher levels of inflammation and higher levels of hypoperfusion. They also had a higher incidence of AKI, shock and more days with IMV than those who did not receive EAT. However, there were no significant differences in all-cause ICU mortality or in the incidence of VAP (Table 1).

### 3.2. Patients with Bacterial Coinfection (COI)

Of the 4197 patients, 654 (15.6%) had a microbiologically confirmed COI. The most frequently isolated microorganisms are shown in Table S1 (Supplementary Materials). A total of 704 microorganisms were isolated from 654 patients. Fifty-four patients (8.2%) and four patients (0.6%) had two and three microorganisms isolated simultaneously. *Streptococcus pneumoniae* (n = 217, 33.2%), methicillin-sensitive *Staphylococcus aureus* (n = 107, 16.4%) and *Pseudomonas aeruginosa* (n = 88, 13.4%) were the most commonly isolated microorganisms.

Twenty-eight patients (4.5%) did not receive EAT despite COI. Patients with COI without EAT had lower severity, lower systemic inflammation and lower hypoperfusion. However, these patients had a higher incidence of VAP and higher mortality than patients with EAT, although this was not significant, possibly due to a type 1 alpha error.(Table 1).

Of the 626 patients with COI and EAT, 85 (13.6%) received IEAT according to microbiological results. The general characteristics of patients with COI and EAT distinguishing appropriate from inappropriate antibiotic treatment are shown in Table 2. Patients with IEAT were older, occurred more frequently during the COVID-19 pandemic period and had a higher incidence of VAP, more days of IMV, longer ICU and hospital stay. The presence of MDR microorganisms was more common in this group and, as expected, a higher crude ICU mortality was observed compared to those who received AEAT.

When patients who did not receive EAT (n = 28) are also considered within inappropriate EAT, a total of 113 (17.3%) patients meet IEAT criteria (global IEAT). The incidence of VAP (36.3% vs. 17.4%, *p* < 0.001) and crude ICU mortality (49.6% vs. 38.4%, *p* = 0.03) were higher in this subgroup compared to those with AEAT.

Of the 654 patients with COI, 135 (20.6%) developed VAP. The characteristics of patients according to the development of VAP or not are shown in Table 3. Strikingly, patients with VAP had lower severity and lower inflammation on ICU admission. The incidence of EAT and crude ICU mortality did not differ between patients with and without VAP. However, AEAT was more common in patients without VAP.

The variables included in the multivariate GLM model for VAP were as follows: AKI, EAT, global IEAT, diabetes, D-dimer, lactate, PCT, CRP, chest X-ray cutoff, and APACHE II according to the significance in Table S3 (Supplementary Materials). Only IEAT (OR = 2.23, 95%CI 1.31–3.73) and chest X-ray cutoff (OR = 1.62, 95%CI 1.07–2.42) were variables associated with the development of VAP (Figure S2, Supplementary Materials).

A total of 264 patients with COI died. Patients who died were older, had a higher degree of severity and inflammation, and had more comorbidities and complications (Table S3 in the Supplementary Materials). However, EAT (96.9% vs. 93.3%, *p* = 0.09) and AEAT (86.2% vs. 80.7%, *p* = 0.07) were not different between survivors and non-survivors. In addition, global IEAT was more common in non-survivors (21.2% vs. 14.6%, *p* = 0.03).

The variables included in the multivariate GLM model for crude ICU mortality were myocardial dysfunction, AKI, EAT, global IEAT, immunosuppression, hematologic disease, chronic heart disease, D.dimer, lactate, PCT, chest X-ray cutoff, GAP-UCI, SOFA, APACHE II and age according to significance in Table S4 (Supplementary Materials). Global IEAT (OR = 1.89, 95%CI 1.13–3.14) but not EAT (OR = 0.58, 95%CI 0.23–1.46) was associated with ICU mortality (Figure S3, Supplementary Materials).

### 3.3. Patients Without Bacterial Coinfection (No-COI)

Of the 3543 patients without COI, 3076 (86.8%) received EAT. Patients with EAT had higher levels of organ dysfunction and inflammation, received more steroids and had a higher incidence of shock on admission compared to patients without EAT. VAP and all-cause mortality in the ICU did not differ between groups (Table 1).

The impact of EAT on outcome can only be causally assessed in a randomised clinical trial. As this is not possible, and in an attempt to address the bias of an observational study, propensity score matching was applied to the non-COI population. For this purpose, the MatchIt package [37] of the R program was used with a “full” method and a caliper of 0.2 (more detailed information on propensity matching can be found in the Supplementary Materials).

After propensity score matching, there was a loss of only 23 patients who could not be matched. Finally, the matched cohort (n = 3520) has 467 controls and 3053 cases receiving EAT. The summary of balance for all data and matched data are shown in Table S5 and in Figures S4 and S5 in the Supplementary Materials.

**Figure 2:**
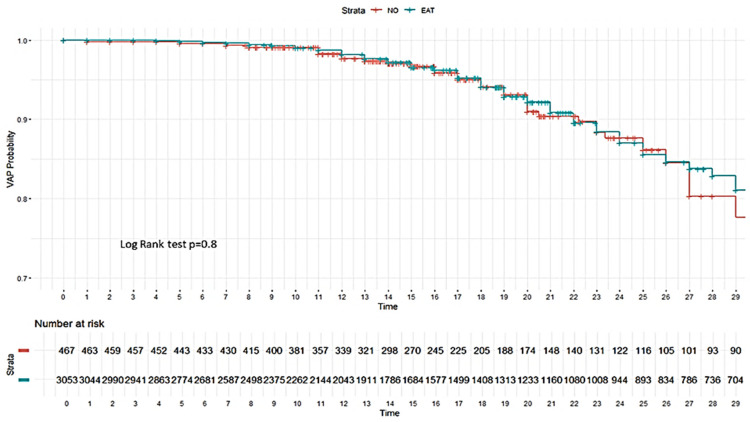
Kaplan–Meier plot for development of ventilator-associated pneumonia (VAP) according to whether or not patients without bacterial co-infection received empiric antibiotic treatment (EAT). As can be seen, there are no significant differences in the probability of developing VAP between the group with EAT (blue line) and the group without EAT (red line) (log rank test *p* = 0.8).

**Figure 3:**
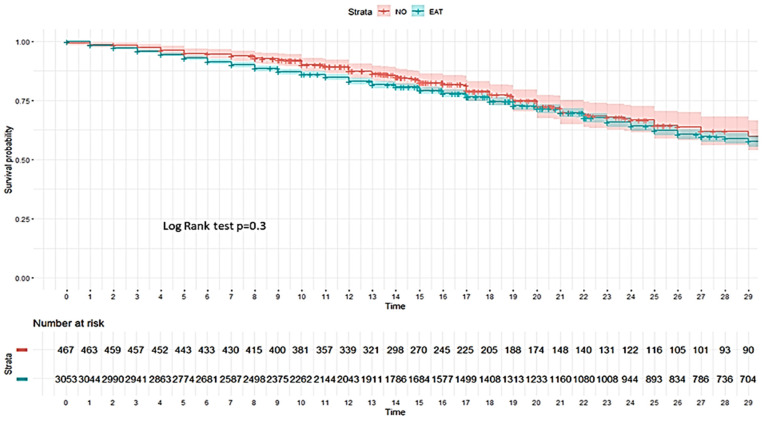
Kaplan–Meier plot for development of all-cause ICU mortality according to whether or not patients without bacterial co-infection received empiric antibiotic treatment (EAT). As can be seen, there are no significant differences in the survival probability between the group with EAT (blue line) and the group without EAT (red line) (log rank test *p* = 0.3).

No impact of EAT was observed on the development of VAP (Figure 2) or on 28-day mortality in the ICU (Figure 3).

### 3.4. Linear Models in Matched Cohort of Patients Without Coinfection

#### 3.4.1. Risk Factors Associated with the Development of Ventilator-Associated Pneumonia (VAP)

The characteristics of patients in the matched cohort according to whether they developed VAP or not are shown in Table S6 in the Supplementary Materials. Patients with VAP had a higher mean age (62 vs. 60; *p* < 0.001) years, a higher degree of hypoperfusion (lactate 2.8 mmol/L vs. 2.2 mmol/L; *p* < 0.001) and a higher incidence of myocardial dysfunction (9.9% vs. 4.8%; *p* < 0.001). In addition, diabetes (17.5% vs. 11.2%; <0.001) and steroid use (71.1% vs. 54.3%; *p* < 0.001) were more common in this group. However, EAT was not associated with VAP on univariate analysis.

**Figure 4:**
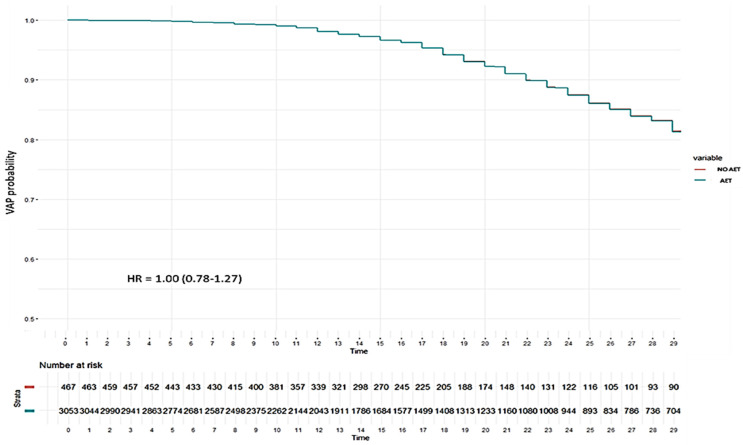
Cox Hazard regression plot for VAP probability according to received empiric antibiotic treatment (EAT) or not in matched cohort of patients without coinfection. As can be seen, the lines are almost superimposed, given that there are no significant differences in the proportional daily risk of developing VAP between the group with (blue line) and without (red line) EAT (HR = 1.0).

There was also no effect of EAT on the proportional hazard of VAP (HR = 1.00, 95%CI 0.78–1.27) when Cox regression was performed adjusting the model for age, chest X-ray cutoff, steroids, diabetes, obesity and lactate with a Shoenfeld global test of 0.35 (Figure 4 and Table S7 in the Supplementary Materials).

EAT was also not associated with the development of VAP in the logistic regression model (OR = 1.02, 95%CI 0.79–1.39). More than 2 infiltrated lung fields (OR = 1.63, 95%CI 1.34–2.0) and steroid administration (OR = 1.97, 95%CI 1.62–2.40) were the variables independently associated with an increased risk of VAP (Figure S6, Supplementary Materials).

#### 3.4.2. Risk Factors Associated with All-Cause ICU Mortality

Of the 3520 patients in the matched cohort, 1192 (33.8%) died. As expected, the deceased patients were older (66 years vs. 58 years, *p* < 0.001), had higher APACHE II (17 vs. 14, *p* < 0.001) and SOFA (7 vs. 6, *p* < 0.001) severity, and higher levels of inflammation. In addition, chronic kidney disease, haematological disease, diabetes and immunosuppression, as well as the presence of AKI, shock and myocardial dysfunction were more common in non-surviving patients (Table S8 in the Supplementary Materials). However, EAT did not appear to be associated with ICU mortality in the univariate analysis.

**Figure 5:**
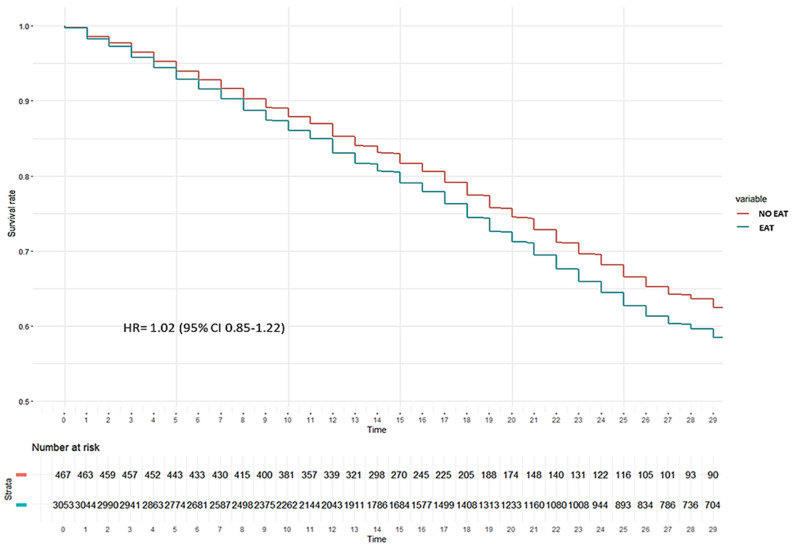
Cox Hazard regression plot for all-cause ICU mortality according to received empiric antibiotic treatment (EAT) or not in matched cohort of patients without coinfection. As can be seen, no significant differences in the proportional daily risk ICU survival between the group with (blue line) and without (red line) EAT was observed (HR = 1.02).

There was also no effect of EAT on the proportional hazard of ICU mortality (HR = 1.02, 95%CI 0.85–1.22) when Cox regression analysis was performed adjusting the model for age, chest X-ray cut-off, AKI, myocardial dysfunction, VAP, steroids, immunodepression, diabetes, haematological disease, chronic kidney disease, shock, D-dimer, lactate, PCT, WBC, LDH, SOFA, APACHEII, GAP_ICU and sex according to significance in univariate analysis. The Schoenfeld global test was *p* < 0.001 (Figure 5 and Table S9 in the Supplementary Materials).

EAT was also not associated with all-cause ICU mortality in the logistic regression model (OR = 1.02, 95%CI 0.81–1.28). (Figure S7, Supplementary Materials).

### 3.5. Non-Linear Analysis–Random Forest Model (RF)

#### 3.5.1. Factors Associated with VAP According to Non-Linear Model

A random forest classifier (RF) model was developed to study the contributions of confounding variables to the dependent variable (VAP) in a non-linear way. All independent variables were included in the RF model and a non-linear relationship with VAP was found. The RF model for VAP had an OOB error rate estimate of 16.0%.

**Figure 6:**
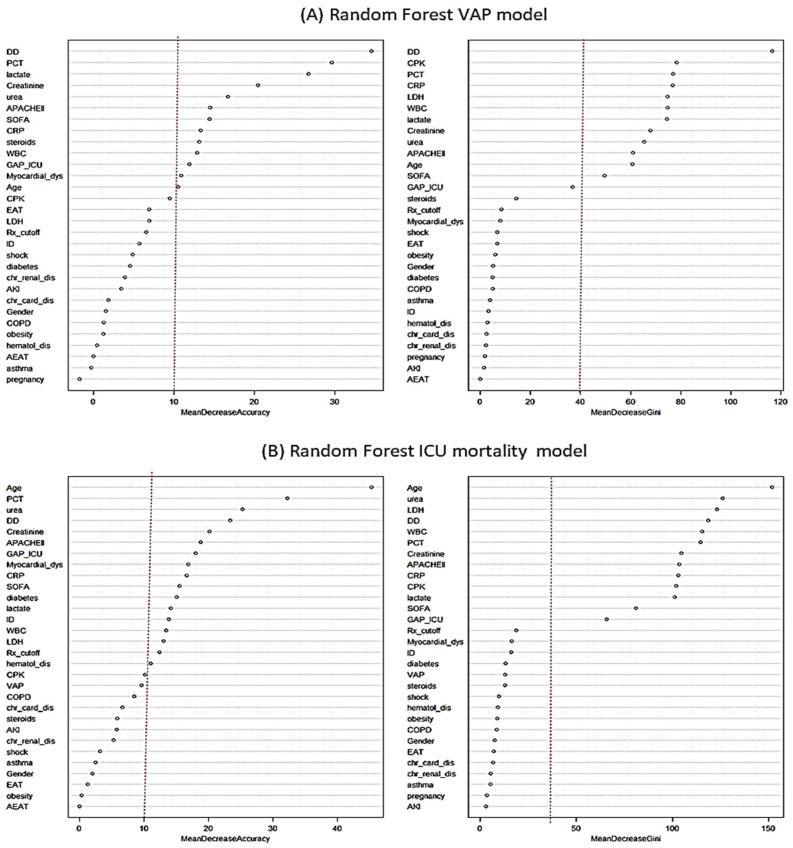
Contribution of each confounding variable according to the random forest (RF) model for variables associated with the development of ventilator-associated pneumonia (VAP) (**A**) and all-cause ICU mortality (**B**). As can be seen in the figure, the empiric antibiotic treatment (EAT) variable is below the cut-off points considered to determine which variables are important in the model (dotted red line) for the development of VAP (**A**) and for ICU mortality (**B**). Abbreviations: cut: cut-off; APACHE II: Acute Physiology and Chronic Health Evaluation; SOFA: Sequential organ failure assessment; EAT: Empiric antibiotic treatment; VAP: ventilator-associated pneumonia; AEAT: Adecuate empiric antibiotic treatment; CPK: creatine phosphokinase; DD: D dímer; WBC: White blood cells; COPD: chronic obstructive pulmonary disease; dis: disfunction; Chr_Card_dis; chronic cardiac disease; AKI: acute kidney injury; CRP:C-reactive protein; GAP_ICU_cut: time elapsed between diagnosing pandemic viral infection and admission to ICU; Chr_renal_dis: Chronic renal disease; ID: immunosuppression; Rx-cutoff: >2 fields with infiltrations in chest X-ray; PCT: procalcitonin; Hematol-dis: Hematologic disease; LDH: Lactate dehydrogenase.

Twelve variables had an impact of more than 10% on the reduction in model accuracy, and twelve variables were associated with a >50% reduction in GINI in the RF model (Figure 6A and Table S8 in the Supplementary Materials). However, AET was not an important variable for VAP development in the RF model.

#### 3.5.2. Factors Associated with All-Cause ICU Mortality According to No-Linear Model

A random forest classifier (RF) model was developed to study the contributions of confounding variables to the dependent variable (non-survivors) in a non-linear way. All independent variables were included in the RF model and non-linear relationship with ICU mortality was found. The RF model for VAP had an OOB error rate estimate of 27.8%.

Seventeen variables had an impact of more than 10% on the reduction in model accuracy, and thirteen variables were associated with a >50% reduction in GINI in the RF model (Figure 6B and Table S9 in the Supplementary Materials). However, AET was not an important variable for all-cause ICU mortality in the RF model.

## 4. Discussion

In this large multicentre cohort of critically ill patients with pandemic viral pneumonia (influenza A[H1N1] and COVID-19), our main conclusion is that empirical antibiotic treatment administered at ICU admission was not associated with a reduction in ventilator-associated pneumonia or ICU mortality in patients without microbiologically confirmed bacterial co-infection. This finding remained consistent after adjusting for confounders using propensity score matching and was confirmed by both traditional multivariate models (Cox and GLM) and non-linear approaches (random forest), reinforcing the robustness of the results.

In contrast, among patients with confirmed bacterial co-infection, EAT was associated with a lower incidence of VAP and ICU mortality, underscoring the importance of timely and appropriate antibiotic administration when bacterial pathogens are present. This highlights a key clinical distinction: while early antibiotics are warranted in patients with confirmed or strongly suspected bacterial infections, their indiscriminate use in all cases of viral pneumonia may be unjustified and potentially harmful.

Evidence regarding the impact of EAT in viral respiratory infections remains limited and heterogeneous [18,23,38,39]. Previous studies have varied widely in terms of population, design, and definitions of co-infection, limiting comparability [19]. Notably, a large international study of 3200 critically ill patients [21] found no effect of EAT on ventilator-associated lower respiratory tract infection, although that analysis did not adjust for baseline differences. A follow-up study evaluating PCT for detecting co-infection also failed to demonstrate a survival benefit from EAT in COVID-19 patients [9]

Other observational studies raise additional concerns. Hovind et al. [40] observed in 3979 patients hospitalized for a viral respiratory infection (influenza virus H3N2, H1N1, influenza B, respiratory syncytial virus [RSV], human metapneumovirus [hMPV] or severe acute respiratory syndrome coronavirus-2 [SARS-CoV-2]) that 67.7% received EAT. When EAT was initiated on admission, it was associated with increased in-hospital mortality (OR = 2.25, 95%CI 1.26–4.02). In addition, patients with EAT had a longer hospital stay. However, the number of critically ill patients in this study is very small (<2%).

Moretto et al. [20] studied the effect of EAT on in-hospital mortality using Cox hazard regression with propensity-matched variable adjustment. Of the 222 patients included, an adverse event (death or ICU transfer) was observed in 60 patients (34%) in the antibiotic group compared with 4 patients (8%) in the no antibiotic group (HR = 2.94 [95%CI: 1.07–8.11]; *p* = 0.04). After propensity score matching, there was no significant association be-tween antibiotic use and outcome (HR = 1.238; 0.77–2.00, *p* = 0.37).

Yin et al. [41], in hospitalized patients with moderate COVID-19, found that during the 30-day follow-up period, 375 (27.3%) of the 1373 patients admitted with non-severe COVID-19 progressed to severe disease. The proportion of patients who progressed to severe COVID-19 was higher in the EAT group compared to the non-EAT group (31.74% vs. 21.94%; *p* < 0.0001). In the Cox model, early antibiotic use was associated with a higher likelihood of progression to severe COVID-19 [aHR = 1.5; 95%CI 1.2–1.9]. After propensity matching, the results remained consistent, showing a higher risk of progression to severe COVID-19 in the EAT group (adjusted HR 1.416, 95%CI 1.069–1.876). Finally, the meta-analysis by Lansbury et al. [19] with over 3800 patients, evidences a low proportion of patients with COVID-19 presenting with COI, whereby the authors conclude that these findings do not support the routine use of antibiotics in the treatment of confirmed COVID-19 infection.

Among critically ill patients, available evidence remains sparse. A small study by Buetti et al. [42] found no benefit of EAT in ICU patients, while a larger study by Saseedharan et al. [38] supported prophylactic antibiotics without comparative data. The study by Wendel–García et al. [18] with a large number of critically ill patients in a Spanish multicentre study concludes, after adjusting covariates by propensity matching, that the administration of EAT is associated with a lower incidence of respiratory superinfection and lower mortality. These conclusions are in contrast to our results, also developed in a Spanish multicentre database, but methodological differences limit comparison. The main difference between the studies is that our population includes not only patients with COVID-19 but also with influenza A (H1N1). Other differences between the studies relate to the inclusion of patients with confirmed fungal infections, the use of broader definitions of respiratory superinfection (including VAP and ventilator-associated tracheobronchitis), and the use of a 24 h time limit from intubation to define empirical antibiotic treatment, which could introduce misclassification bias. In addition, our most inclusive cohort (comprising both SARS-CoV-2 and influenza A [H1N1]) had a higher coinfection rate (15% versus 4%), which is likely to reflect differences in pathogen biology. Notably, influenza A (H1N1) carries a higher risk of bacterial coinfection than SARS-CoV-2, and there are significant differences in the epidemiology of coinfecting bacteria. This could affect prescribing patterns and observed outcomes.

Because viral and bacterial pneumonias share overlapping clinical features and biomarkers, clinicians frequently initiate EAT to avoid undertreatment—an approach widely endorsed by professional societies [5,7,17,35]. Yet, our findings argue for a more selective, evidence-based strategy. Tools such as PCT [9,14,15,24,43] and rapid molecular diagnostics [31,44,45] may help identify true co-infections and enable early antibiotic de-escalation. Notably, PCT performs better in influenza than in COVID-19 [8,9]. Unnecessary broad-spectrum antibiotic use during the COVID-19 pandemic likely contributed to increased antimicrobial resistance [10,11,13,46,47], and over 80% of COVID-19 patients received EAT despite low confirmed co-infection rates [9,19,20,38,40]. Spanish registry data showed VAP incidence more than doubled during the pandemic [48].

Our results strongly indicate that empirical antibiotic treatment should not be used for patients with respiratory infections caused by pandemic viruses in the absence of bacterial co-infection. Therefore, rather than adopting EAT as standard practice, the clinical approach should focus on identifying which patients have COI and would benefit most from antibiotics. Antibiotics should be administered promptly in cases of confirmed or highly suspected bacterial infection, but discontinued early if not justified.

Our study has several limitations. The observational design precludes causal inference. Residual confounding is possible. Data on timing of VAP onset were unavailable, limiting early/late stratification. Differences in co-infection rates and diagnostic approaches across centres may affect generalizability. Nonetheless, the use of robust statistical methods, including propensity score adjustment and machine learning, strengthens the reliability of our findings.

## 5. Conclusions

Our findings suggest that empirical antibiotic treatment should be initiated promptly when there is a high probability of bacterial co-infection. However, empirical antibiotic treatment at ICU admission did not reduce VAP incidence or ICU mortality in critically ill patients with viral pneumonia who lacked confirmed bacterial co-infection. Our findings support a more targeted approach to antibiotic use, guided by microbiology, biomarkers and stewardship principles.

## Figures and Tables

**Table 1:** Characteristics of 4197 ventilated patients included in the study according to diagnosis of bacterial coinfection at ICU admission.

*Variables #*	*Whole Population (n = 4197)*			*Coinfection Patients (n = 654)*			*No Coinfection Patients (n = 3543)*		
	*Total*	*No EAT (n = 495)*	*EAT (n = 3702)*	*Total*	*No EAT (n = 28)*	*EAT (n = 626)*	*Total*	*No EAT (n = 467)*	*EAT (n = 3076)*
General characteristics									
Age, years	60 (49–69)	60 (46–69)	60(49–69)	59 (48–70)	62 (53–69)	59 (48–70)	60 (49–69)	60 (45–69)	60 (49–69) *
Male sex	2746 (65.4)	310 (62.6)	2436 (65.8)	433 (66.2)	19 (67.9)	414 (66.1)	2313	291 (62.3)	2022 (65.7)
APACHE II score	16 (12.21)	15(12–18)	16(12–21) ***	18 (13–24)	14 (10–18)	18 (13–24) ***	15 (12–20)	15 (12–18)	15 (12–21) *
SOFA score	6 (4–8)	5 (4–7)	6 (4–8) ***	7 (5–10)	5 (3–7)	7(5–10) ***	6 (4–8)	5 (4–7)	6 (4–8) ***
Gap-ICU, days	1 (1–3)	2(1–4)	1(1–43) ***	1 (0–2)	1.4 (0–4)	1.0 (0–2)	1 (1–3)	2 (1–4)	1 (1–3) ***
Chest X-ray cutoff	2646 (63.0)	317 (74.9)	2329 (62.9) ***	360 (55.0)	23 (82.1)	337 (53.8) ***	2340 (66.0)	348 (74.5)	1992 (64.8) ***
COVID	2159 (51.4)	279 (56.4)	1880 (50.8) **	191 (29.2)	20 (71.4)	171 (27.3) ***	1968 (55.5)	259 (55.5)	1709 (55.6)
Influenza	2038 (48.5)	216 (43.6)	1822 (49.2) **	463 (70.8)	8 (28.6)	455 (72.7) ***	1575 (44.4)	208 (44.5)	1367 (44.4)
Laboratory									
WBC × 10^3^	8.7 (5.6–13.0)	8.6 (6.4–11.6)	8.8 (5.4–13.1)	8.5 (4.2–13.6)	8.3 (5.3–11.7)	8.6 (4.2–13.7)	8.8 (5.8–12.8)	8.6 (6.5–11.5)	8.8 (5.7–13.0)
LDH U/L	597 (454–763)	600 (487–722)	597 (450–768)	600 (460–745)	556 (467–628)	600 (458–749)	597 (454–766)	600 (490–725)	590 (450–770)
C-RP mg/mL	22.7 (11.5–40.0)	21.1 (9.6–33.0)	23.0 (11.8–42.9) ***	30.2 (16.5–80.4)	13 (6.7–29.0)	31 (17.7–82.5) ***	21.3 (10.8–35.8)	21.4 (10.0–33.1)	21.4 (11.0–37.0)
PCT ng/mL	1.4 (0.30–8.90)	0.84 (0.22–3.35)	1.50 (0.32–10.1) ***	5.3 (1.0–22.3)	0.73 (0.21–2.80)	5.99 (1.2–22.6) ***	1.08 (0.27–3.38)	0.87 (0.22–4.48)	1.14 (0.29–7.11) ***
Creatinine mg/dL	0.92 (0.70–1.32)	0.95 (0.72–1.25)	0.91 (0.70–1.34)	1.1 (0.7–1.8)	0.92 (0.65–1.41)	1.11 (0.77–1.81)	0.90 (0.70–1.26)	0.95 (0.72–1.25)	0.90 (0.70–1.27)
CPK	265 (119–485)	280 (124–487)	263 (119–485)	318 (138–589)	213 (142–358)	326 (138–602)	253 (117–473)	288 (124–497)	248 (115–469)
Lactate mmol/L	2.2 (1.5–3.6)	1.9 (1.4–2.8)	2.3 (1.5–3.7) ***	3.1 (2.0–4.6)	2.0 (1.4–2.5)	3.2 (2.0–4.7) ***	2.1 (1.4–3.9)	1.9 (1.4–2.8)	2.1 (1.4–3.5) **
D-dimer	4343 (1560–8170)	3316 (1360–6200)	4560 (1600–8400) ***	6400 (3030–11,131)	2030 (980–6270)	6585 (3290–11,230) ***	4000 (1470–7620)	3327 (1404–6200)	4111 (1480–7770) ***
Comobidities									
COPD	613 (14.6)	66 (13.3)	547 (14.8)	126 (19.3)	4 (14.3)	122 (19.5)	487 (13.7)	62 (13.3)	425 (13.8)
Asthma	302 (7.2)	37 (7.4)	265 (7.1)	41 (6.3)	2 (7.1)	39 (6.2)	261 (7.3)	35 (7.5)	226 (7.3)
Chr. Heart Dis	271 (6.4)	21 (4.2)	250 (6.7)	57 (8.7)	1 (3.5)	56 (8.9)	214 (6.0)	20 (4.3)	194 (6.3)
Chr.Renal Dis.	260 (6.2)	32 (6.4)	228 (6.1)	52 (7.9)	1 (3.5)	51 (8.1)	208 (5.8)	31 (6.6)	177 (5.7)
Hematologic Dis.	215 (5.1)	27 (5.4)	188 (5.0)	42 (6.4)	2 (7.1)	40 (6.4)	173 (4.8)	25 (5.3)	148 (4.8)
Pregnancy	200 (4.8)	14 (2.8)	186 (5.0) *	53 (8.1)	0 (0.0)	53 (8.4)	147 (4.1)	14 (3.0)	133 (4.3)
Obesity	1471 (35.0)	195 (39.4)	1276 (34.5) *	183 (28.0)	9 (32.1)	174 (27.8)	1288 (36.3)	186 (39.8)	1102 (35.8)
Diabetes	493 (11.7)	83 (16.8)	410 (11.1)	57 (8.7)	5 (17.9)	52 (8.3)	436 (12.3)	78 (16.7)	358 (11.6)
Immunosuppression	349 (8.3)	33 (6.6)	316 (8.5)	77 (11.8)	1 (3.5)	76 (12.1)	272 (7.6)	32 (6.8)	240 (7.8)
Treatments and complications									
Bacterial coinfection	654 (15.6)	28 (5.6)	626 (16.9)	NA	NA	NA	NA	NA	NA
AEAT	541 (12.9)	0 (0.0)	541(86.4)	541 (82.7)	0 (0.0)	541 (86.4)	NA	NA	NA
Corticosteriods	2424 (57.8)	237 (47.9)	2187 (59.1) ***	400 (61.2)	20 (32.1)	380 (60.7)	2024 (57.1)	217 (46.5)	1807 (58.7) ***
VAP	743 (17.7)	88 (17.8)	655 (17.7)	135 (20.6)	10 (35.7)	125 (20.0)	608 (17.1)	78 (16.7)	530 (17.2)
AKI	821 (19.6)	63 (12.7)	758 (20.5) ***	220	6 (21.4)	214 (34.2)	601 (16.9)	57 (12.2)	544 (17.7) **
Myocardial dysfunction	216 (5.1)	19 (3.8)	197(5.3)	131	1 (17.9)	130 (20.8)	201 (5.6)	18 (3.8)	183 (5.9)
Shock	2721 (64.8)	276 (55.8)	2445 (66.0) ***	41	2 (7.1)	39 (6.2)	2223 (62.7)	263 (56.3)	1960 (63.7) **
Outcomes									
LOS ICU, days	16 (9–27)	16 (11 -25)	16 (9–27)	16 (9–29)	19 (12–37)	16 (8–29)	16 (10–27)	16 (11–24)	16 (9–27)
LOS Hospital, days	26 (16–40)	26 (18–35)	26 (16–40)	26 (14–44)	26 (17–47)	26 (14–44)	26 (16–39)	25 (18–35)	26 (16–40)
IMV days	12 (5–22)	8 (1–20)	12 (6.23) ***	13 (7–25)	15 (7–35)	13 (7–25)	12 (5–22)	8 (1–20)	12 (6–22) ***
ICU mortality	1466 (34.9)	159 (32.1)	1307 (35.3)	264 (40.4)	16 (57.1)	248 (39.6)	1202 (33.9)	143 (30.6)	1059 (34.4)

# Continuous variables are shown as median values and percentiles Q1–Q3. Categorical variables are shown as number of cases (n) and percentage (%). (LDH: Lactate dehydrogenase; C-RP: C-reactive protein; CPK: creatine phosphokinase; PCT: procalcitonin; VAP: ventilator associated pneumonia; AKI: acute kidney injury; LOS length of stay; ICU: intensive care units; Gap-ICU: time in days from hospital admission to ICU admission; chest X-ray cutoff: more than 2 lung fields occupied by infiltrates on chest X-ray; MDR: multi-drug resistant bacteria; EAT: empiric antibiotic treatment; AEAT: appropriate empiric antibiotic treatment). For comparisons within each subgroup * *p* < 0.05; ** *p* < 0.01 and *** *p* < 0.001. Primary outcome: All-cause ICU mortality. Secondary outcomes: incidence of ventilator-associated pneumonia (VAP), isolation of multidrug-resistant (MDR) organisms, ICU/hospital length of stay (LOS), duration of mechanical ventilation (IMV), acute kidney injury (AKI), and appropriateness of empirical antibiotic therapy. Exposures: receipt of empirical antibiotic treatment at ICU admission.

**Table 2:** The general characteristics of 626 patients with coinfection (COI) and empiric antibiotic treatment distinguishing appropriate (AEAT) from inappropriate (IEAT) empiric antibiotic treatment.

Variables #	IEAT (n = 85)	AEAT (n = 541)	*p*-Value
**General Characteristics**			
Age, years	62 (56–72)	59 (47–70)	0.009
Male sex	55 (64.7)	359 (66.4)	0.86
APACHE II score	18 (13–21)	19 (14–24)	0.17
SOFA score	7 (5–9)	7 (5–10)	0.04
Gap-ICU, days	1 (1–2)	1 (0–2)	0.18
Chest X-ray cutoff	51 (60.0)	286 (52.9)	0.26
COVID	48 (56.5)	123 (22.7)	<0.001
Influenza	37 (43.5)	418 (77.3)	<0.001
**Laboratory**			
WBC × 10^3^	8.0 (4.9–11.6)	8.7 (3.9–13.9)	0.60
LDH U/L	630 (473–830)	600 (458–745)	0.29
C-RP mg/mL	22.4 (13.0–33.3)	33.4 (19.7–91.3)	<0.001
PCT ng/mL	1.44 (0.24–8.26)	7.86 (1.55–24.0)	<0.001
Creatinine mg/dL	0.87 (0.70–1.48)	1.14 (0.79–1.86)	0.01
CPK	218 (119–399)	338 (151–647)	0.001
Lactate mmol/L	2.3 (1.6–3.6)	2.3 (2.2–4.8)	<0.001
D-dimer	3940 (1179–7200)	6800 (3780–11,700)	
**Comorbidities**			
COPD	11 (12.9)	111 (20.5)	0.13
Asthma	8 (9.4)	31 (5.7)	0.28
Chr. Heart Dis	4 (4.7)	52 (9.6)	0.20
Chr.Renal Dis.	7 (8.2)	44 (8.1)	1.0
Hematologic Dis.	4 (4.7)	36 (6.6)	0.65
Pregnancy	2 (2.3)	51 (9.4)	0.04
Obesity	34 (40.0)	140 (25.9)	0.01
Diabetes	13 (15.3)	39 (7.2)	0.02
Immunosuppression	8 (9.4)	68 (12.6)	0.51
**Treatment and complications**			
Corticosteroids	57 (67.1)	323 (59.7)	0.24
Presence of MDR bacteria	69 (81.2)	98 (18.1)	<0.001
VAP	31 (36.5)	94 (17.4)	<0.001
AKI	20 (23.5)	194 (35.9)	0.03
Myocardial dysfunction	4 (4.7)	10 (1.8)	0.10
Shock	61 (71.8)	424 (78.4)	0.22
**Outcomes**			
LOS ICU, days	22 (12–37)	16 (8–28)	0.001
LOS Hospital, days	30 (21–50)	25 (12–42)	0.008
IMV days	15 (10–30)	12 (6–24)	0.01
ICU mortality	40 (47.1)	208 (38.4)	0.16

# Continuous variables are shown as median values and percentiles Q1–Q3. Categorical variables are shown as number of cases (n) and percentage (%). (LDH: Lactate dehydrogenase; C-RP: C-reactive protein; CPK: creatine phosphokinase; PCT: procalcitonin; VAP: ventilator associated pneumonia; AKI: acute kidney injury; LOS length of stay; ICU: intensive care units; Gap-ICU: time in days from hospital admission to ICU admission; Chest X-ray cutoff: more than 2 lung fields occupied by infiltrates on chest X-ray; MDR: multi-drug resistant bacteria; EAT: empiric antibiotic treatment; AEAT: appropriate empiric antibiotic treatment).

**Table 3:** Patients with bacterial coinfection (COI) according to whether or not they developed ventilator-associated pneumonia (VAP).

Variables #	No VAP (n = 519)	VAP (n = 135)	*p*-Value
**General Characteristics**			
Age, years	59 (47–70)	61 (52–71)	0.09
Male sex	337 (64.9)	96 (71.1)	0.21
APACHE II score	19 (14–25)	17 (12–21)	<0.001
SOFA score	7 (5–10)	7 (4–10)	0.44
Gap-ICU, days	1 (0–2)	1 (0–2)	0.55
Chest X-ray cutoff	268 (51.6)	92 (68.1)	0.001
**Laboratory**			
WBC × 10^3^	8.3 (3.7–13.7)	9.0 (5.0–13.3)	0.64
LDH U/L	600 (450–757)	590 (480–720)	0.62
C-RP mg/mL	33.0 (19.1–85.0)	22.6 (12.6–40.0)	<0.001
PCT ng/mL	7.0 (1.5–24.0)	1.5 (0.4–10.1)	<0.001
Creatinine mg/dL	1.1 (0.7–1.8)	0.9 (0.7–1.4)	0.01
CPK	338 (138–657)	268 (141–400)	0.01
Lactate mmol/L	3.2 (1.2–4.8)	2.3 (1.6–3.7)	<0.001
D-dimer	6667 (3900–11,220)	4000 (1000–9940)	<0.001
**Comorbidities**			
COPD	109 (21.0)	17 (12.6)	0.03
Asthma	32 (6.1)	9 (6.7)	0.98
Chr. Heart Dis	47 (9.0)	10 (7.4)	0.66
Chr.Renal Dis.	41 (7.9)	11 (8.1)	1.00
Hematologic Dis.	34 (6.5)	8 (5.9)	0.94
Pregnancy	46 (8.8)	7 (5.2)	0.22
Obesity	134 (25.8)	49 (36.3)	0.02
Diabetes	34 (6.5)	23 (17.0)	<0.001
Immunosuppression	65 (12.5)	12 (8.9)	0.30
**Treatment and complications**			
Corticosteroids	310 (59.7)	90 (66.7)	0.16
EAT	501 (96.5)	125 (92.6)	0.07
AEAT	451 (86.9)	98 (72.6)	<0.001
Global IEAT	72 (13.9)	41 (30.4)	<0.001
AKI	188 (36.2)	32 (23.7)	0.008
Myocardial dysfunction	10 (1.9)	5 (3.7)	0.20
Shock	403 (77.6)	95 (70.4)	0.09
**Outcomes**			
LOS ICU, days	14 (7–23)	31 (19–48)	<0.001
LOS Hospital, days	23 (12–36)	44 (27–59)	<0.001
IMV days	10 (6–19)	27 (17–41)	<0.001
ICU mortality	208 (40.1)	56 (41.5)	0.84

# Continuous variables are shown as median values and percentiles Q1-Q3. Categorical variables are shown as number of cases (n) and percentage (%). (LDH: Lactate dehydrogenase; C-RP: C-reactive protein: CPK: creatine phosphokinase; PCT: procalcitonin; VAP: ventilator associated pneumonia; AKI: acute kidney injury; LOS length of stay; ICU: intensive care units; Gap-ICU: time in days from hospital admission to ICU admission; Chest X-ray cutoff: more than 2 lung fields occupied by infiltrates on chest X-ray; MDR: multi-drug resistant bacteria; EAT: empiric antibiotic treatment; AEAT: appropriate empiric antibiotic treatment, Global IEAT: includes patients with IEAT and those without EAT).

## Data Availability

The corresponding author (AR) had full access to all the data in the study and takes responsibility for the integrity of the data and the accuracy of the data analysis. All authors approved the final version of the manuscript. The views expressed in this article are those of the authors and not necessarily those of the SEMICYUC. The data supporting the conclusions of this study are available from the Spanish Society of Critical Care (SEMICYUC), but restrictions apply to the availability of these data, which were used under SEMICYUC authorization for the present study and are therefore not publicly available. However, the data can be obtained from the corresponding author (AR) upon reasonable request and with the permission of SEMICYUC.

## Supplementary Materials

The following supporting information can be downloaded at: https://www.mdpi.com/article/10.3390/antibiotics14060594/s1, Figure S1: Flow chart of included patients; Figure S2: Variables associated with the development of ventilator-associated pneumonia (VAP) in multivariate logistic regression model (GLM); Figure S3: Variables associated with the crude ICU mortality in multivariate logistic regression model (GLM); Figure S4: Histograms of propensity scores before and after matching; Figure S5: Plot of mean differences between unadjusted (no matched) and adjusted (matched) covariates; Figure S6: Variables independently associated with VAP in logistic regression model; Figure S7: Variables independently associated with all-cause ICU mortality in logistic regression model; Table S1: Microorganisms isolated (in order of frequency) in 654 patients with bacterial co-infection; Table S2: Patients with COI according to ICU outcome; Table S3: Summary of balance for all data (no-matched) and matched data; Table S4: Characteristics of matched cohort of patients without Coinfection according to ventilator associated pneumonia; Table S5: Variables associated with VAP in the Cox Hazard regression analysis; Table S6: Characteristics of matched cohort patients according to all-cause ICU mortality in patients without bacterial coinfection; Table S7: Variables associated with all-cause ICU mortality in the Cox Hazard regression analysis; Table S8: Importance of variables for VAP according to random forest model; Table S9: Importance of variables for all-cause ICU mortality according to random forest model.
