# Supplementary material for: Does Empirical Antibiotic Use Improve Outcomes in Ventilated Patients with Pandemic Viral Infection? A Multicentre Retrospective Study
Source: Antibiotics (Basel). 2025 Jun 8;14(6):594. doi: 10.3390/antibiotics14060594 (PMC12189047; doi:10.3390/antibiotics14060594)
Supplement: Supplementary file 1 [file antibiotics-14-00594-s001.zip › antibiotics-3685004-supplementary.pdf]

Does Empirical Antibiotic Use Improve Outcomes in Ventilated Patients with Pandemic Viral Infection? A Multicentre Retrospective Study

Supplementary Material

Statistical analysis

First, we performed a descriptive analysis distinguishing between patients with and without empirical antibiotic treatment (EAT) on ICU admission. Continuous vari-ables are presented as median and quantiles (Q1-Q3) and categorical variables as numbers (n) and percentages. Chi-square and U-Mann-Whitney tests were used to compare between groups.

Second, we performed a descriptive analysis differentiating patients with and without the presence of bacterial co-infection (COI). Within each of these subgroups, we differentiated between those with and without EAT.

Third, within the subgroup of patients with COI, we examined the impact of appropriate EAT (AEAT) on mortality, development of VAP, ICU and hospital LOS, and IMV days. For this analysis, patients with IAET were those with IEAT according to microbiological sensitivity and those without AET on ICU admission.

Fourth, within the subgroup of patients without COI, to analyse the impact of EAT on the study objectives, and to convert an observational study into a qua-si-randomized study, a propensity score matching analysis was performed. After matching, the effect of EAT on all cause ICU mortality and on the development of VAP was examined by Kaplan-Meier plot and differences were determined by Log Rang test.

In addition, a Cox proportional hazards (COX) and GLM model was used to de-termine whether EAT was a factor associated with VAP or ICU mortality in multivari-ate adjusted analysis. The results are expressed as hazard ratio (HR) and its 95% confi-dence interval (CI) for COX model and as Odds ratio (OR) and its 95% CI for GLM.

To assess whether the proportional hazard of the Cox model holds, the Schoenfeld residual test was used. The Schoenfeld test uses these residuals to test the proportional hazards hypothesis by examining whether they are correlated over time. If the test is not significant (no correlation), the Schoenfeld residuals are considered to be uncorrelated over time, suggesting that the proportional hazards hypothesis is satisfied and that the effect of the predictor is constant.

Fifth: In addition, to evaluate the impact of EAT on patients without COI, a non-linear regression analysis (Random Forest - RF) was performed to study whether there are non-linear associations between EAT use and crude mortality or the deve-lopment of VAP that cannot be evidenced by linear analysis (GLM). Random forest models are a powerful non-linear tree-based machine learning technique. The developed model was configured to make 500 random trees, with a minimum number of 15 variables per tree. The performance of the RF model was evaluated using out-of-bag (OOB) error. This method allows the prediction error of random forests, boosted decision trees and other machine learning models to be measured using bootstrap aggre-gation. We also plotted the importance of the different variables for the model, which is related to the average loss of accuracy and the Gini index for the classification mo-del. The Gini index is a “measure of disorder”, represented as “MeanDecreaseGini”, which means that the higher the measure, the greater the importance in the generated models, since values close to 0 for the Gini index imply more disorder and

values close to 1 imply less disorder. The higher this measure, the more variability it will contri-bute to the dependent variable. (Figure 1)

Definitions

- Respiratory co-infection (COI) was suspected if a patient presented with signs and symptoms of lower respiratory tract infection, with radiographic evidence of a pulmo-nary infiltrate with no other known cause (23,30,31). Coinfection had to be confirmed by laboratory testing using Centers for Disease Control and Prevention (CDC) criteria . Only respiratory infection microbiologically confirmed with a respiratory spe-cimen or serology obtained within 2 days of ICU admission was considered commu-nity-acquired coinfection. The diagnosis of coinfection was considered "definitive" if respiratory pathogens were isolated from blood or pleural fluid and if serological tests confirmed a fourfold increase of atypical pathogens, including Chlamydia spp., Coxiella burnetti and Moraxella catarrhalis. Only patients with confirmed microbiologic diagnosis were included in the present analysis.
- Ventilator-associated pneumonia (VAP) was defined as a respiratory infection occurring in mechanically ventilated patients according to the guidelines of the European Respiratory Society (ERS), the European Society of Intensive Care Medicine (ESICM), the European Society of Clinical Microbiology and Infectious Diseases (ESCMID), and the Asociación Latinoamericana del Tórax (ALAT). VAP was defined as pneumo-nia occurring more than 48 h after endotracheal intubation with fever, without other apparent causes, with new or increased sputum production, positive endotracheal as-pirate (ETA) culture (>10^6 CFU/mL), or bronchoalveolar lavage (BAL) culture (>10^4 CFU/mL), with at least one respiratory pathogen known to cause pneumonia, and with radiographic evidence of nosocomial pneumonia.
- Empirical antibiotic treatment (EAT) were selected based on specialist clinical judgment and internal ICU protocols, which could subsequently be modified by the ASP (antimicrobial stewardship program) team, based on the clinical response in the days following VAP diagnosis or final microbiology results.
- Appropriate empiric antibiotic treatment (AEAT): Was defined as the administration of an antibiotic on admission to the ICU before the microbiological results are available and adjusted to the susceptibility of the pathogen when the microbiological results are available. AEAT was de-termined by the attending physician in each centre.
- Inappropriate empirical antibiotic treatment (IEAT): Was defined as antibiotic treatment administered on admission to the ICU that was not adapted to the susceptibility of the pathogen when microbiological results are available. In addition, the use of antibiotics at ICU admission in patients with no bacterial co-infection was also included in this definition.
- Multi-drug-resistant bacteria (MDR) are defined as those isolated strains that are not sensitive to at least one agent from three families of antimicrobials.
- Acute Kidney injury (AKI): The diagnosis of AKI was considered according to the Acute Kidney Injury Network (AKIN) described in the international KDIGO guidelines.
- GAP-UCI: was defined as the time elapsed between diagnosing pandemic viral infection and admission to ICU.
- GAP-Diagnosis: Was defined as the period of time between the onset of clinical symptoms and the microbiological diagnosis of the pandemic viral infection.
- Immunosuppression: this variable includes patients with active solid organ cancer, chemotherapy and patients on steroid therapy with a dose of prednisone > 30 mg/day or equivalent on prolonged therapy.

- Shock: was defined as any patient with noradrenaline requirements at a dose > 0.1 mcg/kg/min during the first hours of ICU admission.
- Chest x-ray cutoff: more than 2 lung fields occupied by infiltrates on chest x-ray

Figure S1: Flow Chart of included patients . (IMV: invasive machanical ventilation; COI: coinfection; EAT: empiric antibiotic treatment; VAP: ventilator-associated pnuemonia; AEAT: appropriate empiric antibiotic treatment; IEAT: inappropriate empiric antibiotic treatment; ICU: intensive care unit)

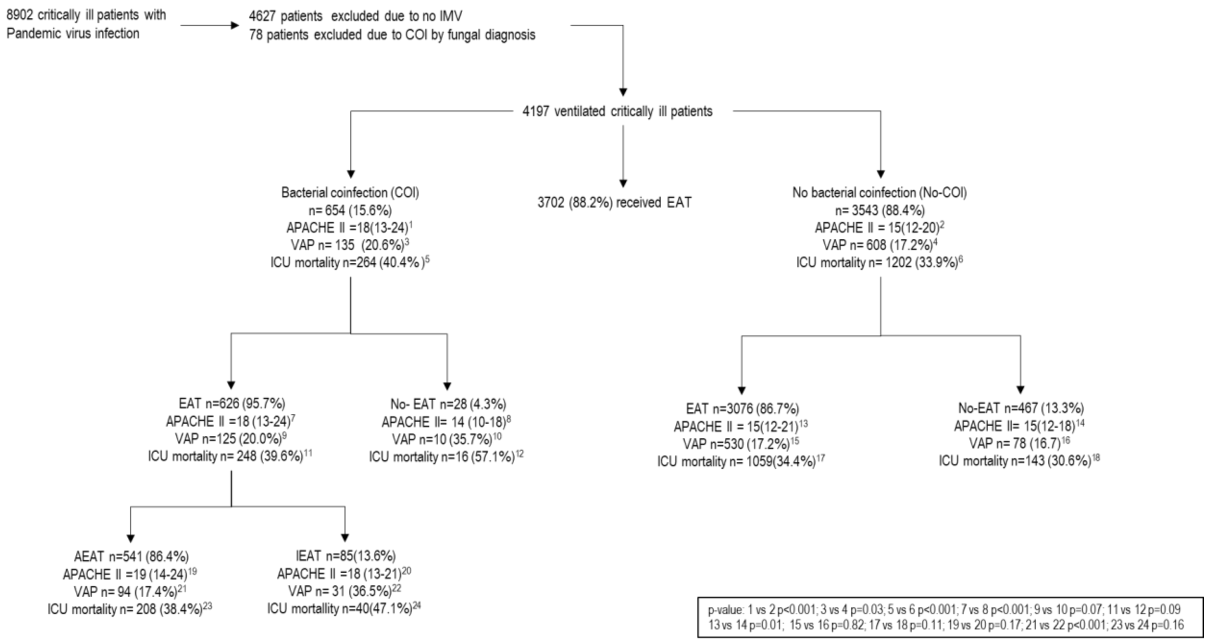

Table S1: Microorganisms isolated (in order of frequency) in 654 patients with bacterial co-infection (note that 54 patients (8.2%) had 2 microorganisms isolated and 4 (0.6%) had 3 microorganisms isolated simultaneously).

Microorganisms isolates	n (%)*
Streptococcus pneumoniae	217 (33.2)
Methicillin-sensitive *Staphylococcus aureus*	107 (16.4)
Pseudomonas aeruginosa	88 (13.4)
Klebsiella spp.	47 (7.2)
Haemophilus influenzae	41 (6.3)
Streptococcus pyogenes	41 (6.3)
Methicillin-resistant Staphylococcus aureus	35 (5.3)
Escherichia coli	31 (4.7)
Acinetobacter baumannii	15 (2.3)
Serratia spp.	15 (2.3)
Stenotrophomonas malthophilia	13 (2.0)
Enterobacter spp.	8 (1.2)
Moraxella catarrhalis	7 (1.1)
Chlamydia pneumoniae	5 (0.8)
Legionella pneumophilia	5 (0.8)
Mycoplasma pneumoniae	5 (0.8)
Citrobacter spp.	4 (0.6)
Coxiella burnetii	3 (0.4)
Morganella morganii	3 (0.4)
Streptococcus agalactiae	3 (0.4)
Proteus spp.	3 (0.4)
Neisseria pneumoniae	3 (0.4)
Others	5 (0.8)
Total	704

* percentages are considered over the total number of patients.

Figure S2: Variables associated with the development of ventilator-associated pneumonia (VAP) in multivariate logistic regression model (GLM).

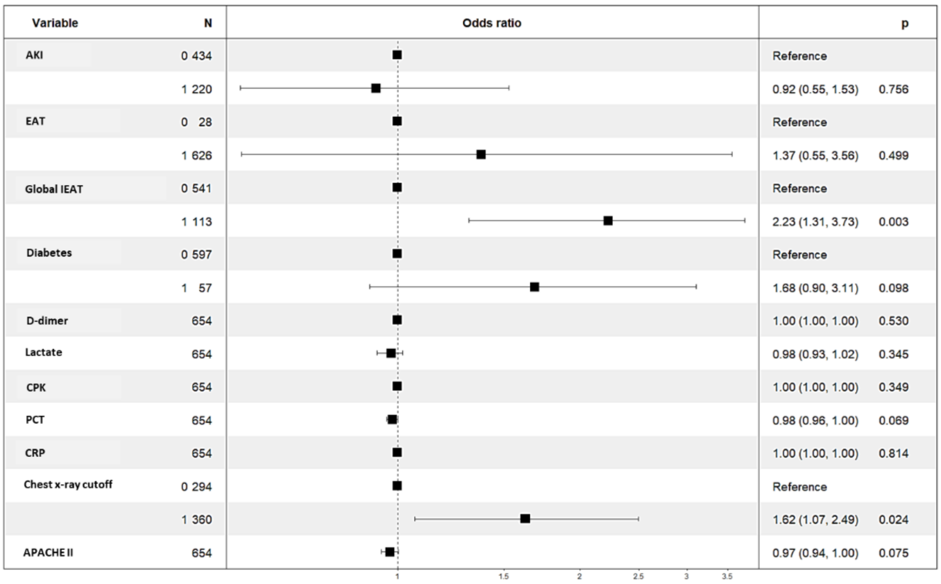

(CRP: C-reactive protein: CPK: creatine phosphokinase; PCT: procalcitonin; AKI: acute kidney injury; Chest x-ray cutoff: more than 2 lung fields occupied by infiltrates on chest x-ray; EAT: Empiric antibiotic treatment; Global IEAT: global inappropriate empiric antibiotic treatment include patients with IEAT plus patients without EAT)

Table S2: Patients with COI according to ICU outcome.

Variables	*Survivors (n=390)*	*Non-survivors (n=264)*	*p-value*
	General Characteristics		
Age, years	57 (47-68)	63 (52-74)	<0.001
Male sex	257 (65.9)	176 (66.7)	0.90
APACHE II score	18 (13-23)	20 (15-26)	<0.001
SOFA score	7 (5-9)	8 (5-10)	0.002
Gap-ICU, days	1 (0-2)	1 (0-3)	<0.001
Chest x-ray cutoff	200 (51.3)	160 (60.6)	0.02
	Laboratory		
WBC x10^3	8.5 (4.7-13.5)	8.4 (3.5-14.0)	0.51
LDH U/L	560 (456-720)	620 (670-770)	0.14
C-RP mg/mL	29.0 (16.0-76.0)	32.2 (18.1-84.5)	0.24
PCT ng/mL	4.12 (0.83-19.7)	8.26(1.32-24.4)	0.002
Creatinine mg/dL	1.0 (0.74-1.59)	1.25 (0.86-2.0)	<0.001
CPK	320 (140-600)	315 (140-570)	0.81
Lactate mmol/L	2.9 (2.0-4.14)	3.5 (2.10-5.35)	0.01
D-dimer	5600 (2600-9540)	7340 (3760-13,560)	<0.001
	Comorbidities		
COPD	79 (20.3)	47 (17.8)	0.49
Asthma	28 (7.2)	13 (4.9)	0.31
Chr. Heart Dis	22 (5.6)	35 (13.3)	0.001
Chr.Renal Dis.	24 (6.1)	28 (10.6)	0.05
Hematologic Dis.	14 (3.6)	28 (10.6)	0.001
Pregnancy	32 (8.2)	21 (7.9)	1.0
Obesity	109 (27.9)	74 (28.0)	1.0
Diabetes	30 (7.7)	27 (10.2)	0.32
Immunosuppression	31 (7.9)	46 (17.4)	<0.001
	Treatment and complications		
Corticosteroids	223 (57.2)	177 (67.0)	0.01
EAT	378 (96.9)	248 (93.9)	0.09
AEAT	336 (86.2)	213 (80.7)	0.07
Global IEAT *	57 (14.6)	56 (21.2)	0.03
VAP	79 (20.3)	56 (21.2)	0.84
AKI	107 (27.4)	113 (42.8)	<0.001
Myocardial dysfunction	2 (0.5)	13 (4.9)	0.001
Shock	290 (74.4)	208 (78.8)	0.22
	Outcomes		
LOS ICU, days	19 (12-34)	11 (5-23)	<0.001
LOS Hospital, days	33 (22-49)	14 (6-27)	<0.001
IMV days	14 (8-27)	11 (4-22)	<0.001

#Continuous variables are shown as median values and percentiles Q1-Q3. Categorical variables are shown as number of cases (n) and percentage (%). (LDH: Lactate dehydrogenase; CRP: C-reactive protein: CPK: creatine phosphokinase; PCT: procalcitonin, VAP: ventilator associated pneumonia; AKI: acute kidney injury, LOS length of stay, ICU: intensive care units; Gap-ICU: Time in days from hospital admission to ICU admission; Chest x-ray cutoff: more than 2 lung fields occupied by infiltrates on chest x-ray; MDR: multi-drug resistant bacteria ; * Global IEAT: include patients with IEAT plus patients without EAT)

Figure S3: Variables associated with the crude ICU mortality in multivariate logistic regression model (GLM).

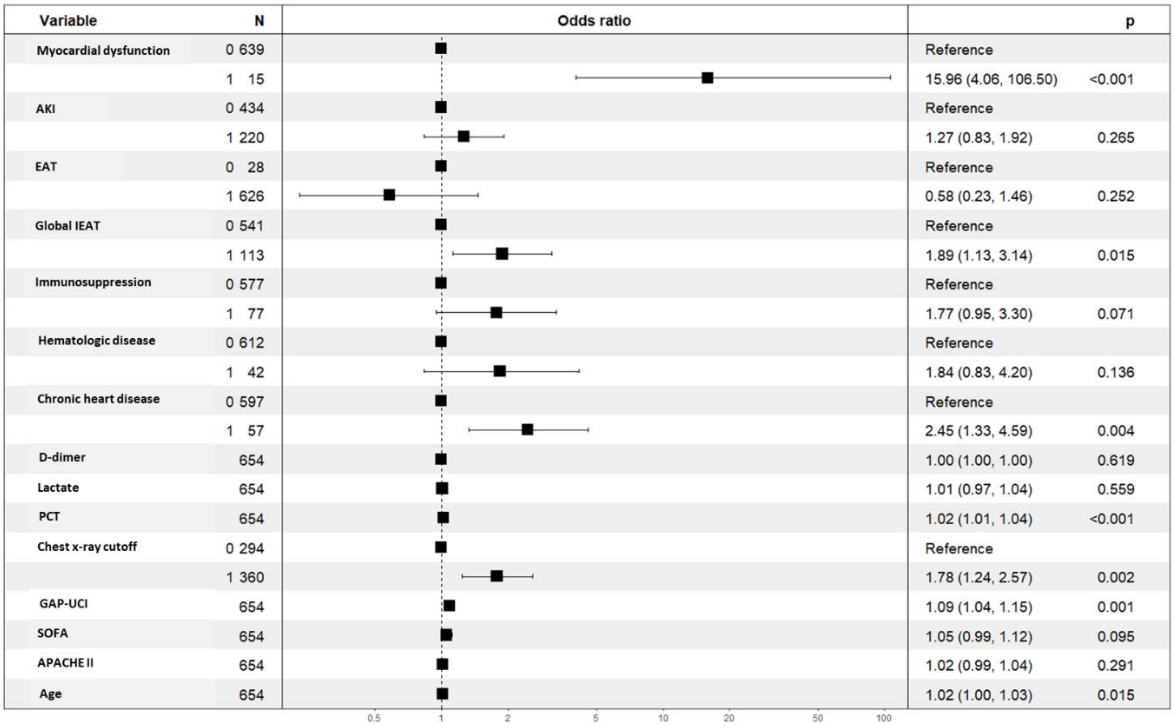

(AKI: acute kidney injury; EAT: empiric antibiotic treatment ; PCT: procalcitonin; Gap-ICU: Time in days from hospital admission to ICU admission; Chest x-ray cutoff: more than 2 lung fields occupied by infiltrates on chest x-ray; Global IEAT: include patients with IEAT plus patients without EAT)

Propensity Score Matching

In an attempt to address the bias of an observational study and to adjust for different covariates between groups receiving and not receiving EAT, we performed propensity score matching using the 'MatchIt' package of the 'R' statistical programme (Ho, D. E., Imai, K., King, G., Stuart, E. A. MatchIt: Nonparametric Preprocessing for Parametric Causal Inference. Journal of Statistical Software, 2011;42(8). doi:10.18637/jss.v042.i08).

MatchIt provides a simple and straightforward interface for covariate balancing in observational studies using Mahalanobis distance matching with substitution and balance assessment. We have implemented the "Full" method for optimal full matching with a caliper of 0.2, which is the width of the calipers to be used in the matching. It should be a numerical vector with each value named according to the variable to which the caliper applies. For positive values, the distance between the paired units must not be greater than the caliper provided; for negative values, the distance between the paired units must be greater than the absolute value of the caliper provided.

After propensity score matching, there was a loss of only 23 patients who could not be matched. Finally, the matched cohort has 467 controls without EAT and 3053 cases receiving EAT. The summary of balance for all data and matched data are show in Table S5

Table S3: Summary of balance for all data (no-matched) and matched data

Table S5

Summary of Balance for All Data

	Means Treated <chr>	Means Control <chr>	Std. Mean Diff. <chr>	Var. Ratio <chr>	eCDF Mean <chr>	eCDF Max <chr>
distance	0.8717	0.8453	0.5073	0.8509	0.1380	0.2233
Gender0	0.3427	0.3769	-0.0721	.	0.0342	0.0342
Gender1	0.6573	0.6231	0.0721	.	0.0342	0.0342
Age	58.5217	56.5096	0.1435	0.7814	0.0262	0.0704
GAP_ICU	2.5405	2.8617	-0.0855	1.5391	0.0955	0.2359
APACHEII	16.7395	15.8594	0.1189	1.4079	0.0454	0.1034
SOFA	6.5269	5.8387	0.2301	1.3276	0.0753	0.1369
Rx_cutoff0	0.3524	0.2548	0.2043	.	0.0976	0.0976
Rx_cutoff1	0.6476	0.7452	-0.2043	.	0.0976	0.0976
PCT	6.5431	4.6518	0.1326	1.5148	0.0595	0.1038
DD	7856.7859	6357.8477	0.0624	3.4398	0.0476	0.1167
steroids0	0.4125	0.5353	-0.2494	.	0.1228	0.1228
steroids1	0.5875	0.4647	0.2494	.	0.1228	0.1228
AKI0	0.8231	0.8779	-0.1436	.	0.0548	0.0548
AKI1	0.1769	0.1221	0.1436	.	0.0548	0.0548
shock0	0.3628	0.4368	-0.1540	.	0.0740	0.0740
shock1	0.6372	0.5632	0.1540	.	0.0740	0.0740

Summary of Balance for Matched Data

	Means Treated <chr>	Means Control <chr>	Std. Mean Diff. <chr>	Var. Ratio <chr>	eCDF Mean <chr>	eCDF Max <chr>	Std. Pair Dist. <chr>
distance	0.8720	0.8720	0.0008	1.0001	0.0018	0.0121	0.0089
Gender0	0.3416	0.3438	-0.0045	.	0.0021	0.0021	0.9214
Gender1	0.6584	0.6562	0.0045	.	0.0021	0.0021	0.9214
Age	58.5155	60.8277	-0.1649	1.0657	0.0366	0.1110	1.0384
GAP_ICU	2.4860	2.7513	-0.0706	1.5335	0.0958	0.1964	0.7784
APACHEII	16.6895	17.2528	-0.0761	1.0492	0.0501	0.0983	0.9739
SOFA	6.5106	6.2740	0.0791	1.0616	0.0259	0.0632	0.8540
Rx_cutoff0	0.3518	0.4261	-0.1556	.	0.0744	0.0744	0.7654
Rx_cutoff1	0.6482	0.5739	0.1556	.	0.0744	0.0744	0.7654
PCT	6.3828	5.7892	0.0416	1.3046	0.0468	0.0942	0.5339
DD	7840.1666	7097.0687	0.0309	2.5951	0.0348	0.0901	0.3877
steroids0	0.4107	0.4058	0.0100	.	0.0049	0.0049	0.7174
steroids1	0.5893	0.5942	-0.0100	.	0.0049	0.0049	0.7174
AKI0	0.8248	0.8012	0.0618	.	0.0236	0.0236	0.6417
AKI1	0.1752	0.1988	-0.0618	.	0.0236	0.0236	0.6417
shock0	0.3626	0.4392	-0.1594	.	0.0766	0.0766	0.8748
shock1	0.6374	0.5608	0.1594	.	0.0766	0.0766	0.8748

Figure S4: Histograms of propensity scores before and after matching**.**

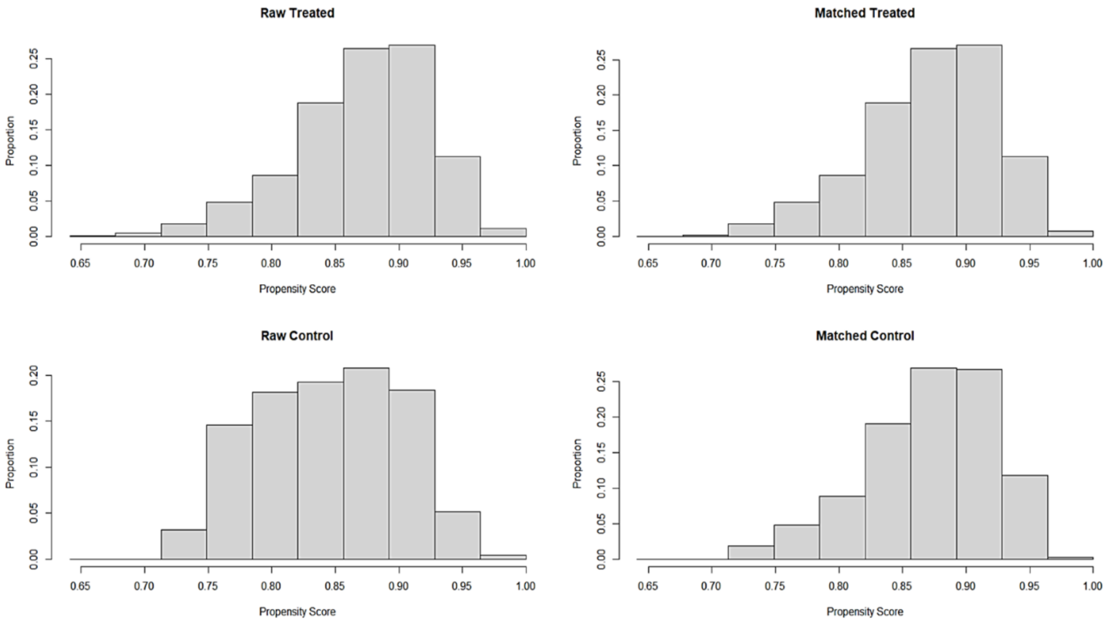

Figure S5: Plot of mean differences between unadjusted (no matched) and adjusted (matched) covariates

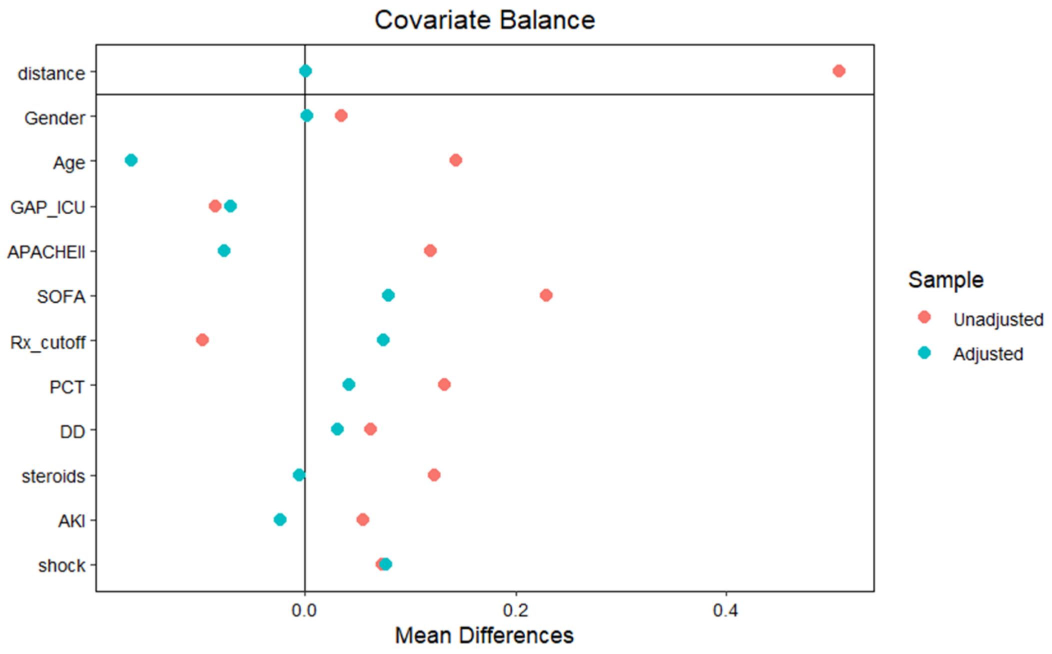

Table S4 : Characteristics of matched cohort of patients without Coinfection according to ventilator associated pneumonia.

Variables	*No VAP (n=2916)*	*VAP (n=604)*	*p-value*
	General Characteristics		
Age, years	60 (48-69)	62 (52-71)	<0.001
Male sex	1879 (64.4)	422 (69.9)	0.01
APACHE II score	15 (12-20)	15 (12-20)	0.51
SOFA score	6 (4-8)	6 (4-8)	0.32
Chest x-ray cutoff	1878 (64.4)	449 (74.3)	<0.001
	Laboratory		
WBC x10^3	8.7 (5.7-12.2)	9.0 (6.0-13.0)	0.09
LDH U/L	600 (450-770)	590 (450-740)	0.46
C-RP mg/mL	22.0 (11.2-38.5)	18.0 (9.8-29.7)	<0.001
PCT ng/mL	1.26 (0.30-7.30)	0.56 (0.20-2.05)	<0.001
Creatinine mg/dL	0.90 (0.70-1.27)	0.90 (0.70-1.22)	0.31
CPK	260 (117-480)	230 (116-440)	0.08
Lactate mmol/L	2.2 (1.5-3.5)	2.8 (1.2-2.9)	<0.001
D-dimer	4200 (1650-7690)	2200 (750-7190)	<0.001
	Comorbidities		
COPD	401 (13.8)	81 (13.4)	0.87
Asthma	219 (7.5)	42 (6.9)	0.69
Chr. Heart Dis	184 (6.3)	29 (4.8)	0.18
Chr.Renal Dis.	171 (5.9)	34 (5.6)	0.89
Hematologic Dis.	142 (4.9)	26 (4.3)	0.62
Pregnancy	126 (4.3)	17 (2.8)	0.62
Obesity	1040 (35.7)	242 (40.1)	0.04
Diabetes	328 (11.2)	106 (17.5)	<0.001
Immunosuppression	229 (7.8)	38 (6.3)	0.21
	Treatment and complications		
Corticosteroids	1583 (54.3)	433 (71.7)	<0.001
EAT	2527 (86.7)	526 (87.1)	0.83
AKI	496 (17.0)	96 (15.9)	0.54
Myocardial dysfunction	141 (4.8)	60 (9.9)	<0.001
Shock	1856 (63.6)	353 (58.4)	0.01
	Outcomes		
LOS ICU, days	14 (9-23)	30 (20-46)	<0.001
LOS Hospital, days	24 (15-35)	40 (27-60)	<0.001
ICU mortality	953 (32.7)	239 (39.6)	0.001

#Continuous variables are shown as median values and percentiles Q1-Q3. Categorical variables are shown as number of cases (n) and percentage (%). (LDH: Lactate dehydrogenase; CRP: C-reactive protein: CPK: creatine phosphokinase; PCT: procalcitonin, VAP: ventilator associated pneumonia; AKI: acute kidney injury, LOS length of stay, ICU: intensive care units; Gap-ICU: Time in days from hospital admission to ICU admission; Chest x-ray cutoff: more than 2 lung fields occupied by infiltrates on chest x-ray; MDR: multi-drug resistant bacteria ; * Global IEAT: include patients with IEAT plus patients without EAT)

Table S5: Variables associated with VAP in the Cox Hazard regresssion analysis.

	HR	95 % CI	
EAT	1.0046	0.7898	1.2777
Age	1.0046	0.9980	1.0112
Rx_cutoff	1.2632	1.0514	1.5177*
Steroids	1.2934	1.0776	1.5524**
Diabetes	0.9475	0.7590	1.1827
Obesity	1.1317	0.9596	1.3346
Lactate	0.9646	0.9404	0.9893**

Signif. codes: 0 '***' 0.001 '**' 0.01 '*' 0.05 '.' 0.1 ' ' 1

Concordance= 0.58(se = 0.015)
Likelihood ratio test= 36.27 on 7 df,p=6e-06
Wald test = 31.51 on 7 df,p=5e-05
Score (logrank) test = 32.1 on 7 df,p=4e-05

Figure S6: Variables independently associated with VAP in logistic regression model

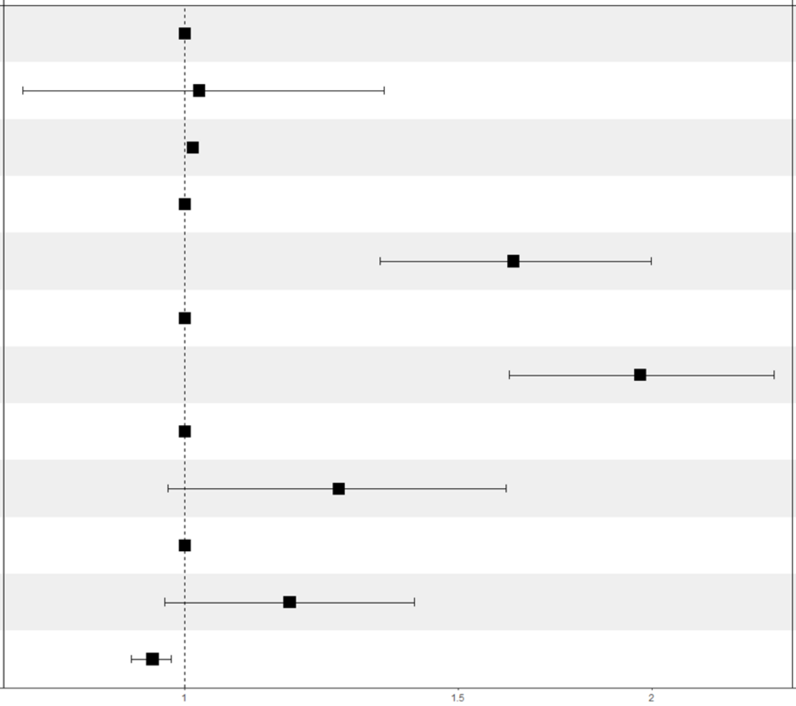

Variable		N	Odds ratio	p
EAT	0	467	Reference	
	1	3053	1.02 (0.79, 1.34)	0.874
Age		3520	1.01 (1.01, 1.02)	<0.001
Rx_cutoff	0	1193	Reference	
	1	2327	1.63 (1.34, 2.00)	<0.001
steroids	0	1504	Reference	
	1	2016	1.97 (1.62, 2.40)	<0.001
diabetes	0	3086	Reference	
	1	434	1.26 (0.98, 1.61)	0.073
obesity	0	2238	Reference	
	1	1282	1.17 (0.97, 1.41)	0.098
lactate		3520	0.95 (0.92, 0.98)	0.002

Table S6: Characteristics of matched cohort patients according to all cause ICU mortality in patients without bacterial coinfection

Variables	*Survival (n=2328)*	*Non-survival (n=1192)*	*p-value*
	General Characteristics		
Age, years	58 (46-66)	66 (55-73)	<0.001
Male sex	1466 (63)	835 (70.1)	<0.001
APACHE II score	14 (11-19)	17 (13-23)	<0.001
SOFA score	6 (4-8)	7 (5-9)	<0.001
Chest x-ray cutoff	1470 (63.1)	857 (72.0)	<0.001
GAP-UCI	1 (1-3)	2 (1-4)	0.01
	Laboratory		
WBC x10^3	8.5 (5.6-12.1)	9.5 (6.2-14.2)	<0,001
LDH U/L	586 (435-750)	620 (490-796)	<0.001
C-RP mg/mL	21.0 (10.8-36.0)	22.0 (11.0-35.4)	0.48
PCT ng/mL	1.02 (0.26-5.13)	1.25 (0.30-9.0)	<0.001
Creatinine mg/dL	0.86 (0.70-1.16)	1.01 (0.77-1.45)	<0.001
CPK	250 (115-470)	257 (120-480)	0.87
Lactate mmol/L	2.0 (1.4-3.1)	2.3 (1.5-3.8)	<0.001
D-dimer	3700 (1300-6800)	4900 (1700-9300)	<0.001
	Comorbidities		
COPD	300 (12.9)	182 (15.3)	0.05
Asthma	185 (8.0)	76 (6.4)	0.10
Chr. Heart Dis	125 (5.4)	88 (7.4)	0.02
Chr.Renal Dis.	111 (4.8)	94 (7.9)	<0.001
Hematologic Dis.	71 (3.0)	97 (8.1)	<0.001
Pregnancy	109 (4.7)	34 (2.8)	0.01
Obesity	854 (36.7)	428 (36.0)	0.67
Diabetes	233 (10.0)	201 (17.0)	<0.001
Immunosuppression	119 (5.1)	148 (12.4)	<0.001
	Treatment and complications		
Corticosteroids	1259 (54.1)	757 (63.5)	<0.001
EAT	2004 (86.1)	1049 (88.0)	0.12
VAP	365 (15.7)	239 (20.1)	0.001
AKI	305 (13.1)	287 (24.1)	<0.001
Myocardial dysfunction	75 (3.2)	126 (10.6)	<0.001
Shock	1400 (60.1)	809 (68.0)	<0.001
	Outcomes		
LOS ICU, days	17 (11-30)	14 (7-23)	<0.001
LOS Hospital, days	30 (20-46)	18 (9-28)	<0.001

Table S7 : Variables associated with all cause ICU mortality in the Cox Hazard regresssion analysis.

	HR	95% CI	
EAT1	1.0262	0.8595	1.2252
Age	1.0210	1.0159	1.0260***
Rx_cutoff1	1.2633	1.1120	1.4352***
AKI1	1.2069	1.0319	1.4117*
Myocardial_dys1	1.6750	1.3808	2.0319***
VAP1	0.5187	0.4463	0.6027***
steroids1	1.1060	0.9785	1.2501
ID1	1.5711	1.2789	1.9301***
diabetes1	1.2156	1.0349	1.4278*
hematol_dis1	1.2721	0.9949	1.6266
chr_renal_dis1	0.8252	0.6547	1.0401
shock1	1.0595	0.9307	1.2062
DD	1.0000	1.0000	1.0000**
lactate	1.0073	0.9961	1.0186
PCT	1.0087	1.0068	1.0107***
WBC	1.0009	0.9992	1.0026
LDH	1.0003	1.0001	1.0004*
SOFA	1.0235	1.0002	1.0473***
APACHEII	1.0249	1.0156	1.0343*
GAP_ICU	1.0169	1.0031	1.0309***
Gender1	1.0359	0.9131	1.1751

Signif. codes: 0 ‘***’ 0.001 ‘**’ 0.01 ‘*’ 0.05 ‘.’ 0.1 ‘ ’ 1

Concordance= 0.698 (se = 0.009)
Likelihood ratio test= 448.7 on 21 df,   p=<2e-16
Wald test   = 507.2 on 21 df,   p=<2e-16
Score (logrank) test = 546.9 on 21 df,   p=<2e-16

Figure S7: Variables independently associated with all cause ICU mortality in logistic regression model

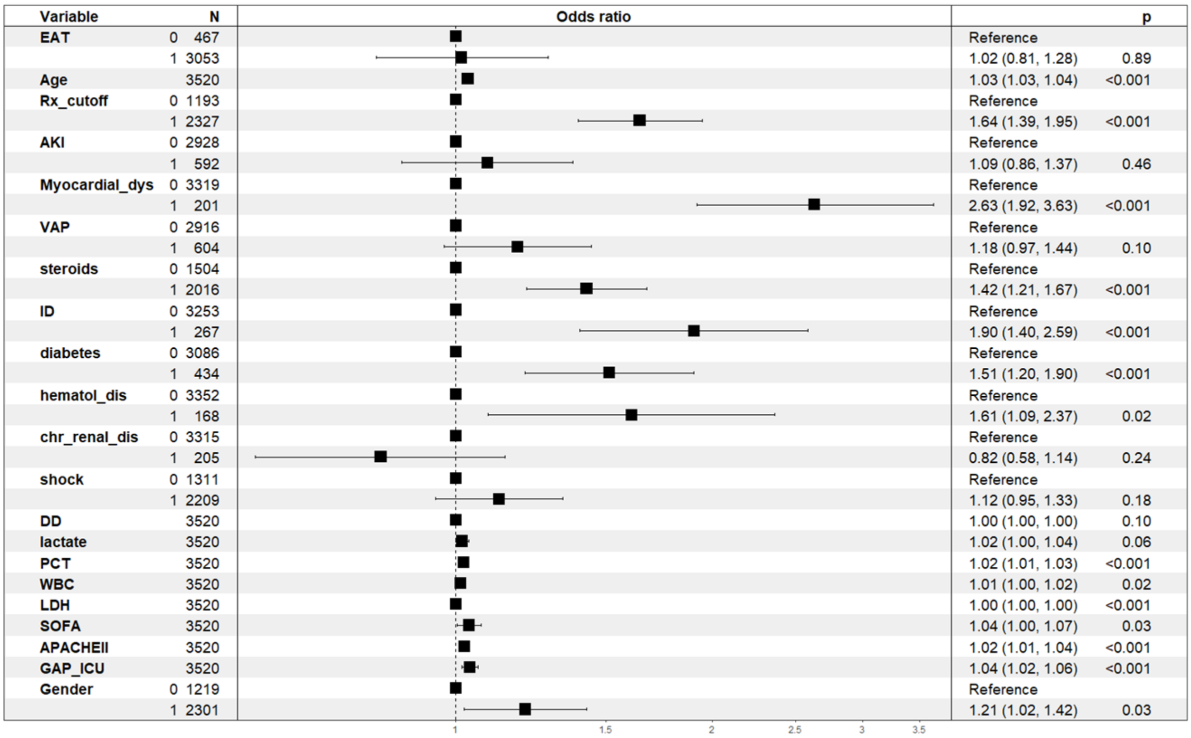

Table S10: Importance of variables for VAP according to Random Forest model

	0	1	MeanDecreaseAccuracy	MeanDecreaseGini
Gender	2.34	-1.51	1.51	5.26
Age	9.81	3.06	10.51	60.73
GAP_ICU	9.85	6.14	11.92	36.86
APACHEII	15.33	0.90	14.51	60.99
SOFA	15.43	-0.47	14.42	49.68
LDH	5.76	3.86	6.91	74.80
CPK	10.22	-0.83	9.46	78.34
WBC	12.39	2.41	12.89	74.76
Creatinine	22.44	-5.07	20.47	67.96
urea	18.13	-2.98	16.70	65.36
CRP	15.58	-7.01	13.31	76.78
PCT	29.01	-2.85	29.64	77.03
lactate	26.38	0.40	26.74	74.57
DD	28.06	18.89	34.56	116.39
shock	4.90	0.30	4.87	6.92
asthma	-0.16	-0.35	-0.28	4.04
COPD	1.07	0.61	1.28	4.88
chr_card_dis	2.15	-0.45	1.82	2.46
chr_renal_dis	4.90	-2.13	3.90	2.37
hematol_dis	1.63	-2.88	0.45	2.93
pregnancy	-1.22	-1.69	-1.73	1.89
obesity	0.11	2.09	1.20	6.04
diabetes	3.48	2.45	4.56	4.98
ID	6.06	0.44	5.71	3.24
steroids	6.33	16.23	13.15	14.36
EAT	5.62	4.39	6.92	6.79
AEAT	0.00	0.00	0.00	0.00
Myocardial_dys	8.28	6.98	10.88	8.12
AKI	4.11	-3.15	3.42	1.45
Rx_cutoff	3.89	8.12	6.55	8.37

Table S11: Importance of variables for all cause ICU mortality according to Random Forest model

	0	1	MeanDecreaseAccuracy	MeanDecreaseGini
Gender	3.04	-0.70	1.94	7.65
Age	36.89	21.67	45.31	151.75
GAP_ICU	17.46	5.93	17.99	65.83
APACHEII	13.84	12.01	18.79	103.61
SOFA	6.54	14.75	15.47	81.05
LDH	12.88	4.32	13.06	123.04
CPK	13.92	-3.19	10.11	101.83
WBC	17.98	-2.39	13.39	115.47
Creatinine	19.84	-0.66	20.12	104.58
urea	22.36	5.19	25.30	125.97
CRP	19.75	-5.74	16.65	103.08
PCT	31.81	0.20	32.22	114.59
lactate	14.41	2.37	14.12	101.06
DD	25.19	0.12	23.32	118.67
shock	-2.32	7.13	3.16	9.74
asthma	2.23	1.03	2.43	5.49
COPD	12.31	-3.94	8.46	8.65
chr_card_dis	9.45	-3.06	6.61	6.84
chr_renal_dis	9.86	-5.75	5.23	5.50
hematol_dis	8.39	6.20	11.01	9.15
pregnancy	-0.16	-0.99	-0.78	3.43
obesity	-0.05	0.59	0.29	8.80
diabetes	13.69	6.70	15.06	13.15
ID	9.27	9.92	13.84	16.18
steroids	6.13	1.68	5.85	12.83
EAT	0.74	1.03	1.29	6.93
AEAT	0.00	0.00	0.00	0.00
VAP	5.69	8.34	9.62	13.01
Myocardial_dys	13.67	10.18	16.86	16.37
AKI	6.22	-2.87	5.78	2.92
Rx_cutoff	12.49	4.64	12.41	18.85
